# Supplementary material for: Are carbon emissions trading and green financial instruments synergistic? -Comprehensive quantitative research based on content analysis
Source: PLoS One. 2024 Mar 7;19(3):e0298601. doi: 10.1371/journal.pone.0298601 (PMC10919601; doi:10.1371/journal.pone.0298601)
Supplement: S1 Data — (ZIP) [file pone.0298601.s001.zip › Data/地级市Z_F工作报告.zip!/░▓╗╒/┬φ░░╔╜╩╨/└┤╫╘╣1⁄2╓┌║┼í╛┬φ┐╦╩2╛▌═°í┐_2005.docx]

# **政府工作报告（2005）**

【字体大小：[大](http://zwgk.mas.gov.cn/openness/detail/content/javascript:doZoom(18)) [中](http://zwgk.mas.gov.cn/openness/detail/content/javascript:doZoom(15)) [小](http://zwgk.mas.gov.cn/openness/detail/content/javascript:doZoom(14))】

政  府  工  作  报  告

——2005年1月9日在马鞍山市第十三届人民代表大会第四次会议上

市   长   姚玉舟

各位代表：

现在，我代表市人民政府，向大会作工作报告，请予审议，并请市政协委员和其他列席人员提出意见。

一、2004年政府工作回顾

刚刚过去的2004年，是我市全面实施“两个率先”战略并取得显著成绩的一年。一年来，我们在省委、省政府和市委的领导下，以邓小平理论和“三个代表”重要思想为指导，认真贯彻党的十六大和十六届三中、四中全会精神，按照科学发展观的要求，全面理解、积极落实中央宏观调控的决策部署，坚持加快发展不动摇，凝心聚力，排难而进，胜利完成了市十三届人大三次会议确定的各项目标任务，提前一年基本实现“十五”计划主要目标。

（一）国民经济持续快速增长，主要指标创历史新高。全年完成生产总值261亿元（预计，下同），同比增长19.2%，人均生产总值跨上2万元新台阶。财政收入在2003年突破30亿元的基础上再创佳绩，完成45.8亿元，增长47%，其中三个城区和开发区财政收入均超过亿元，当涂县财政收入突破4亿元。全社会固定资产投资完成145亿元，增长35.8%，其中工业技改投资65亿元，15个重点工业项目建成发挥效益，投资结构更趋合理，发展后劲明显增??工业克服市场波动、生产要素供应趋紧等不利因素影响，增速不断加快，效益显著提升。全市规模以上工业企业增至264户，当年新增80户；规模以上工业实现增加值150亿元，增长35%；销售收入达405亿元，增长52%；综合效益指数235，比上年提高40个百分点；实现利税76亿元，增长42%。马钢产能跃上800万吨钢新台阶，实现销售收入260亿元，分别净增200万吨钢和100亿元；“十一五”发展建设规划获得国家批准，前期工作取得重要进展。华菱公司实现向重型汽车生产企业的跨越，山鹰公司销售收入突破10亿元，达12亿元。县区工业蓬勃发展，规模以上工业产值和销售收入分别增长42%和45%。建筑业市场开拓能力增强，完成增加值18亿元，增长10%。认真贯彻中央1号文件精神，出台并落实促进农民增收的16条政策措施，在全省率先免征农业税及其附加，农业和农村经济发展形势喜人。全年实现农业增加值17.9亿元，增长10.5%。粮食播种面积和总产量均接近历史最好水平，水稻、油菜优质品率进一步提高，水产生态养殖取得新成效，优势农产品生产基地建设全面启动。农业规模化经营步伐加快，承包土地50亩以上的种粮大户增至815户，承包面积占全市粮食播种面积的17%。产业化龙头企业牵动作用明显增强，直接受益农民达16.5万人。第三产业快速发展，实现增加值63.4亿元，增长12.2%。商业市场稳中见旺，物价基本稳定。全年完成社会消费品零售总额52.6亿元，增长12.3%。旅游宣传和市场开拓取得积极成效，马钢被确定为首批国家工业旅游示范点，全市旅游业总收入增长35%。房地产开发完成投资22亿元，竣工面积90万平方米，商品房销售额增长65.7%，较好地满足了城乡居民的住房需求。金融业保持稳健运行态势，年末本外币存款余额263.2亿元，贷款余额180.4亿元，分别比年初净增53.8亿元和28.1亿元。保险、证券、运输、信息咨询等服务业继续发展。

（二）对外开放实现新突破，民营经济发展势头强劲。继续把招商引资作为经济工作的重中之重，组织开展赴广东、山东、西安、香港、欧美、日韩等重点招商活动，成功举办吟诗节经贸洽谈会，强势推进招商引资。全年实际利用外资突破1亿美元，达1.1亿美元，实际利用内资达38亿元，分别增长35.3%和57.6%，呈现出单个项目投资增大、科技含量增加、产业集中度增强、加工制造业项目增多的新特点。农业招商引资规模扩大、领域拓宽，共引进项目186个，到位资金3.3亿元。开发区基础设施不断完善，招商引资主力军作用日益显现。全年实际利用外资6850万美元，占全市总额的62.5%；续建、新建工业项目40个，22个项目竣工投产；完成规模以上工业总产值26亿元，增长30%。对外贸易和经济合作取得新成绩。全市完成进出口总额9亿美元，其中出口1.7亿美元，增长41.4%。马钢在澳大利亚参股设立铁矿合营企业，十七冶境外工程中标额达2.5亿元。更加主动地参与区域经济合作，加大融入长三角工作力度，与上海、杭州等城市进行了广泛的高层交流。参与承办“南京重洽会”，与都市圈内外城市就一批重大项目进行了洽谈对接。与芜湖、铜陵在推进人才开发一体化发展等方面取得实质性进展。和陕西省宝鸡市建立了友好城市关系，在工业、农业等领域达成合作协议。对外宣传工作力度加大，城市知名度明显提高。

把培育民营经济作为增强自主增长能力的重要举措来抓，进一步放宽投资领域、降低准入门槛、改革登记方式、实行告知承诺制，民营经济保持快速发展的良好势头。全年新增个体工商户6109户；新增民营企业1200户，增长14%；纳税额达3.3亿元，增长69%。民营经济经营领域加速拓展，工业型、科技型企业比重持续上升，外向度进一步提高，为促进全市经济发展、扩大就业和增加居民收入作出了积极贡献。

（三）各项改革向纵深推进，市场化机制进一步完善。把2004年作为深化改革年，多渠道筹措改革成本，强力实施中小企业改革扫尾攻坚。全年完成企业改革57户，提前两年实现省政府确定的中小企业改革目标。通过改革，一批成长型企业步入良性发展轨道，成为新的经济增长点。剥离企业办社会职能取得新突破，马钢18所中小学和退休人员管理实现平稳移交。江东公司改革取得重大进展，马鞍山钢铁设计院成功改制为股份制公司，矿山研究院以及供电公司、烟草公司、三二二地质队等驻马单位内部改革继续深化。山鹰公司股票增发成功，再融资4.24亿元，实现净资产翻番。国有资产管理体制改革深入推进，出资人制度进一步完善，市产权交易所正式挂牌运营。按照有利发展、因事制宜、分类指导的原则，制定完成生产经营类事业单位转企改制工作实施意见，事业单位人事和分配制度改革取得积极成效。完成新一轮乡镇区划调整，全市乡镇由31个调整为19个。行政审批制度改革全面推进，再次取消、调整行政审批事项42项。财政管理体制改革取得新进展，当涂县14个乡镇实现乡财县管。集中开展食品、药品、农资等市场专项整治活动，市场经济秩序进一步规范。根据国务院统一部署，认真开展土地市场治理整顿，规范经营性用地“招拍挂”行为，及时查处土地违法违规案件，国土资源管理工作得到加强。住房制度改革深入实施，公积金归集使用工作取得新成绩。其它各项改革正积极稳妥地向前推进。

（四）城乡建设管理力度加大，综合服务功能明显增强。大力推进新区建设和旧城改造，全年完成城市建设投资40.6亿元，比上年增长28%。建成湖西南路、湖南东路、慈湖河路、霍里山大道等14条骨干道路，新增城市道路总里程26.8公里，东环路以内的路网骨架基本形成，采西桥建成通车，城市向东向南发展的构架进一步拉开。公用事业加快发展，天然气利用一期工程顺利完工，第四水厂加紧建设，电力、邮政、电信等服务功能进一步增强。积极创建国家环保模范城市，第二污水处理厂建成投入运行，山鹰垃圾焚烧发电综合利用工程并网发电，向山垃圾无害化处理工程开工建设，一批重点工业污染治理项目顺利完成。小城镇建设力度加大，共完成投资5.6亿元，当涂县城和中心镇服务功能明显增强。农村交通建设步伐加快，当高大桥竣工通车，全年建成公路140公里。长江大桥项目被列入国家长江干流桥梁建设规划，前期工作取得实质性进展。水利工程和农田水利基本建设完成投资2亿元，城乡防洪保安能力和农业生产条件得到较大改善。

市容市貌继续改观。投资1.1亿元对雨山湖进行综合整治，截污、清淤、游道驳岸和亮化美化工程全部竣工，水体质量显著改善，“城市明珠”焕发新的光彩。建成佳山、雨山公园和采石延园，建成区绿化覆盖率提高到42.8%。改造背街小巷道路35条、老旧小区7个，历时三年、投入6000多万元的背街小巷和老旧小区综合整治工程基本完成，成片危旧房改造工程启动实施。集中拆除违法建筑18.3万平方米，环境卫生管理水平进一步提高。开展道路交通秩序专项整治，在全省率先达到B类城市二等优秀管理水平。“生态马鞍山”建设取得积极进展，被省政府批准为生态省建设首批综合示范基地，成为全省唯一的国家清洁生产试点城市，生态市建设规划正在加紧编制。

（五）科技教育取得重要成果，社会事业协调健康发展。完成国家863新材料成果产业化基地发展规划编制，新材料产业园主体园区开工建设。高新技术创业服务中心功能进一步完善，入驻企业40户，后续工程建设启动实施。产学研合作领域加速拓展，与清华大学签订全面合作协议，与北京科技大学签订共建国家863新材料成果产业化基地框架协议，企业与高等院校和科研院所的科技合作关系得到加强。当涂县被列为全国科技工作试点县，雨山区荣获全国科普示范城区称号。马钢实施科研攻关项目128项，其中国家级4项、省级7项。高新技术产业和民营科技企业加速发展，全年高新技术产业实现产值80亿元，增长26.5%。教育事业发展步伐加快。市二中迁址新建工程如期完成，师范附小和珍珠园小学被批准为省特色示范小学。安工大顺利通过教育部本科教学水平评估，师范学校升格为师范高等专科学校，网络大学一期工程投入使用。安徽工贸技师学院主体工程按期建成，市职教中心被列为省重点建设示范性职业学校。股份制的成功学校注册成立，全市新增民办学校6所。文化事业继续发展。中国国际吟诗节获得圆满成功，大型灯展等群众文化活动受到广泛赞誉。专业文艺创作取得丰硕成果，黄梅戏儿童音乐剧《男子汉行动》进京演出引起强烈反响。群众文化活动广泛开展，幸福广场被评为全国特色文化广场。公共卫生基础设施建设力度加大，市疾病预防控制中心和紧急救援中心基本建成，传染病专科医院开工建设。新型农村合作医疗制度在当涂县启动实施，覆盖率达83.8%。去年初，面对突如其来的高致病性禽流感疫情，及时启动应急预案，采取果断措施扑灭疫情，取得了防治斗争的重大胜利。群众体育运动蓬勃开展，成功举办国际太极拳交流大会，竞技体育取得优异成绩。人口和计划生育工作继续加强，低生育水平保持稳定。统计工作迈上新台阶，第一次全国经济普查工作扎实开展。积极实施名牌战略，质量兴市工作取得新成效。广播电视宣传质量不断提高，安全播出落到实处。“星光计划”工程基本完成。档案事业健康发展。续修志书工作稳步推进。民族宗教工作有新的提高。其它各项社会事业全面进步。

（六）改善民生工作成效显著，城乡居民生活水平大幅提升。坚持以人为本，高度重视解决困难群众的生产生活问题。把促进就业再就业摆在突出位置来抓，全面落实各项扶持政策，全年新增就业2.8万人，其中失业人员再就业1.7万人，“4050”人员再就业3527人；城镇登记失业率控制在3.8%，就业再就业工作受到国务院和省政府表彰。积极扶持创业和组织起来就业，在三区分别建成创业服务中心，开辟两个创业园和两条再就业一条街。认真做好再就业培训工作，开办再就业培训电视课堂，建立农民职业培训学校，全年培训人数达1.7万人。着力提高劳务输出组织化程度，18个乡镇建立了劳动保障事务所，在沿海大中城市设立10个联系点，农村新-family: 仿宋_GB2312">2.2万人。强化养老、失业、医疗保险扩面征缴和基金监管，覆盖面继续扩大，基金征缴率均在99%以上。工伤、生育保险启动实施，五大保险制度全部建立，社会保险体系进一步完善。企业退休人员移交社区管理工作积极推进。城市低保工作实现动态管理、分类施保，农村特困群众生活救助机制开始启动。切实保障被征地农民合法权益，严格依法开展土地征迁，各项补偿及时足额到位。针对工作中存在的问题，积极研究建立长效机制，制定被征地农民养老保障制度，加快安置房建设步伐，力求从根本上解决被征地农民的后顾之忧。继续实施廉租房制度，对1486户住房困难的城镇低保户实行住房补贴。消除了1373户农村特困户人居草房，市郊1.3万农民饮用自来水困难即将得到解决，年初安排的10件为民办实事项目顺利完成。在经济发展的基础上，全面提高企业退休人员养老金、失业人员失业保险金、退养人员基本生活费、劳动者最低工资标准和城市低保标准，城镇职工工资水平大幅提高。城市居民人均可支配收入突破1万元，达10188元，增长15.9%；农民人均纯收入3910元，增长25.2%；城乡居民人均储蓄在全省率先突破1万元，达11500元。

（七）文明创建活动扎实开展，民主法制和政府自身建设得到加强。全面启动全国文明城市创建活动，各项工作稳步向前推进。积极创建“八百里皖江文明长廊”，文明单位、文明社区等基层创建工作取得明显成效。认真贯彻《公民道德建设实施纲要》，广泛开展“做新时期合格公民”主题教育活动，弘扬和培育民族精神，市民文明素质进一步提高。大力整治校园周边环境，积极倡导文明健康的课余活动，未成年人思想道德建设继续加强。认真落实与清华大学共建学习型城市示范工程协议，学习型政府建设取得新进展。创建全国双拥模范城市活动全面展开，国防动员和民兵预备役建设迈出新步伐，人防工作扎实推进。加大法制宣传力度，“四五”普法活动深入开展。第二届社区换届和首次社区直选试点工作顺利完成，厂务、村务公开制度得到较好落实。坚持“严打”方针，严厉打击杀人、抢劫等暴力性犯罪和黑恶势力，一批影响恶劣的重大刑事案件及时告破。着力推进社会治安防控体系建设，社会治安形势保持稳定。全面落实安全生产责任制和责任追究制，加大安全隐患治理投入，集中开展以非煤矿山为重点的专项整治，有效扼制了重特大安全事故的发生。强化信访工作责任制，实行领导干部下访督查制度，开展对信访突出问题的排查调处，努力化解社会不稳定因素。坚持并完善市政府负责同志信访接待日制度，全年52次接待信访群众900多人次，一批群众关心、关注的热点难点问题得到有效解决。

一年来，市政府坚持向市人大报告工作、向市政协通报情况制度，自觉接受市人大法律监督、工作监督和市政协民主监督，政府系统承办的4件人大代表议案、105件建议批评意见和325件政协提案如期办复。市政府负责同志先后12次约请、走访市人大代表和政协委员，通报全市经济社会发展情况，听取代表、委员的意见和建议。加强与各民主党派、工商联以及工会、共青团、妇联等人民团体的联系，注重发挥舆论和社会的监督作用。进一步细化政府工作规则，规范政府系统运作程序，行政效能继续提高。积极改进目标管理办法，激励作用明显增强。政务公开全面推行，新闻发布制度正式建立，电子政务建设取得新进展。认真落实廉政建设责任制，执法监察工作力度加大，违法违纪案件得到严肃查处。强化审计监督，全面实行国库集中支付制度，继续规范政府采购工作，注重从源头上治理腐败。

各位代表，过去的一年非同寻常。在这一年里，面对宏观经济环境的新变化，我们牢固树立科学发展观和正确政绩观，审时度势，未雨绸缪，果断决策，变挑战为机遇，牢牢把握加快发展的主动权；针对经济社会生活中出现的新矛盾，坚持用改革和创新的办法，沉着应对，扎实工作，破解难题，变压力为动力，奋起开创现代化建设的新局面。成绩的取得，凝聚着全市人民的智慧和汗水，倾注了各行各业一大批同志的辛劳和奉献，得益于方方面面的关心和帮助。在此，我代表市人民政府，向辛勤工作在各条战线上的广大干部群众，向给予政府工作大力支持的市人大代表、市政协委员、各民主党派、工商联和无党派人士、各人民团体，向参与、支持马鞍山现代化建设的中央和省驻马单位、解放军和武警官兵、海内外朋友，表示衷心的感谢并致以崇高的敬意！

在肯定成绩的同时，我们也清醒地认识到，在加快发展的道路上还有许多困难和问题，群众还有不满意的地方，政府工作还存在一些缺点和不足之处。一是与先进发达地区相比，我市经济总量还有较大差距，赶超的任务十分艰巨；产业结构性矛盾和经济外向度偏低问题仍然比较突出，经济竞争能力和抗风险能力有待加强；部分生产要素供给偏紧，已经成为制约经济加快发展的重要因素。二是随着经济转轨和社会转型的进一步深入，发展中的一些矛盾比较尖锐地表现出来，生态环境不容乐观，维护稳定的任务相当繁重，就业再就业压力仍然较大，城镇低收入群众生活还比较困难，保持农民收入稳定增长的基础不够牢固。三是政府职能转变还不能完全适应改革发展的要求，依法行政水平有待提高，少数部门和工作人员作风不实、服务意识不强的问题还不同程度存在，发展环境需要继续改善。对此，我们将予以高度重视，在今后的工作中努力应对和解决。

二、2005年发展目标及主要经济工作

2005年是全面超额完成“十五”计划、衔接“十一五”发展的重要一年，也是加快实现“两个率先”目标的关键之年。做好今年工作，意义十分重大。当前，加快发展的形势总体趋好。世界经济温和复苏的态势没有改变，经济全球化和区域经济一体化步伐继续加快，国际资本和产业向我国转移的趋势依然强劲。中央继续加强和改善宏观调控，实行稳健的财政政策和货币政策，合理调控总量，着力调整结构，政策环境将有利于经济平稳较快增长。经过多年坚持不懈地深化改革、扩大开放、加大投入，我市发展的动力、活力和后劲明显增强，尤其是广大干部群众思想更加解放，精神更加振奋，经验更加丰富，这些都是我们做好今年工作的条件所在、信心所在。我们又必须看到，经济社会发展中的不确定因素依然较多，一些深层次的矛盾和问题短期内难以根本消除，周边城市强劲发展态势给我们带来巨大的竞争压力。我们必须保持清醒头脑，兴利除弊，积极进取，努力实现经济社会全面协调可持续发展。

今年政府工作的总体要求是：以邓小平理论和“三个代表”重要思想为指导，认真贯彻党的十六大和十六届三中、四中全会精神，全面落实科学发展观，继续深化改革和扩大开放，扎实推进结构调整和经济增长方式转变，着力解决经济社会生活中的突出矛盾和问题，不断提高人民生活水平，确保全面和超额完成“十五”各项目标任务，为加速实现“两个率先”目标奠定坚实基础。2005年全市经济社会发展主要预期目标是：生产总值增长12%；财政收入增长12.5%；全社会固定资产投资增长15%以上；实际利用外资1.2亿美元，实际利用内资40亿元；外贸出口增长17%；社会消费品零售总额增长10%；城镇居民人均可支配收入、农民人均纯收入分别增长8%和7%；城镇登记失业率控制在4.2%以内；人口出生率控制在11.5‰以内。

实现今年的经济发展目标，重点要做好以下工作：

（一）强力实施大开放主战略，推动外向型经济和民营经济更快更大发展。继续把扩大开放作为推动经济发展的主要动力，进一步营造大招商、大开放格局，全面提高对外对内开放水平。

千方百计提升引资规模和质量。不断改进招商方法，以项目为抓手，以开发园区为载体，致力引进一批牵动性强的高新技术产业、先进制造业、能源交通和农业产业化项目，推动产业结构升级，增强经济发展后劲。加强项目跟踪落实，突出抓好一批重大引资项目的资金到位和开工建设，提高项目履约率。进一步强化“亲商、安商、富商”意识，努力提供优质高效服务，鼓励外资企业增资扩股，吸引更多外商来我市投资兴业。加快建立与国际接轨的行政管理模式和市场运行机制，下决心解决一批影响投资环境的突出问题，积极构建良好的政务环境和诚信、规范、有序的市场秩序，争创招商引资新优势。

加快推进开发园区建设和发展。进一步完善园区发展规划，科学设置项目入区、入园标准，全面提升园区建设发展水平。按照“全面建成西区、加快建设东区、积极拓展南区”的要求，继续推进开发区基础设施建设，为现有企业发展和新项目入区提供良好条件。开发区要继续发挥招商引资主力军作用，加大项目引进力度，力争在引进大项目上取得新突破，全年实际利用外资7000万美元、内资6亿元以上。全面加快在谈、签约、在建项目实施进度，充分发挥竣工项目的投资效应，确保全区工业总产值增长50%以上。

努力扩大对外经贸和区域经济合作。积极落实国家鼓励出口的优惠政策，实施出口市场多元化战略，加大对机电产品、高新技术产品出口促进力度，确保进出口总额突破10亿美元，其中出口1.9亿美元以上。加快“走出去”步伐，推动外经企业扩大对外工程承包和劳务合作。高度重视上海世博会、南京全运会带来的商机，加强与长三角城市的合作交流，全方位参与南京都市圈建设，积极拓展与芜湖、铜陵及其他友好城市的合作领域，在推进区域合作中加快发展。

全力推动民营经济发展实现新飞跃。继续优化民营经济发展环境，建立和完善面向中小企业的社会化服务体系，帮助民营企业解决发展中遇到的困难和问题。充分发挥各类创业载体作用，大力营造全民创业氛围，推动民营经济快速发展。全年力争新增个体工商户6000户、民营企业1300户，纳税额超4亿元。引导民营企业加快体制创新和管理创新，鼓励民营企业以多种形式聚集各类优质要素，下大气力培育一批骨干民营企业，力争全年新增销售收入超3000万元的民营企业10户以上。

（二）深入推进各项改革，建立健全全面协调可持续发展的制度保障。坚持社会主义市场经济改革方向，不断消除制约经济社会发展的体制性障碍，为加快发展注入强大的动力和活力。

继续深化企业改革。巩固中小企业改革成果，加强提留资产监管，妥善处理企业改革遗留问题。引导改制企业完善法人治理结构，健全现代企业制度，实现企业的规范运作和健康发展。鼓励企业进入资本市场融资，推进天源科技公司上市步伐，加快培育境内外上市资源。积极引进战略投资者参与重点骨干企业改革重组，支持驻马单位深化产权制度改革，切实抓好山鹰集团公司规范化改制。继续推进马钢主辅分离，加快实施辅业改制，不断壮大非钢产业。建立健全国有资产监管体制和运营体系，着力盘活经营性存量资产，实现国有资产保值增值。

分类推进事业单位改革。进一步完善事业单位改革各项政策措施，在生产经营类事业单位率先启动实施，争取年内完成转企改制任务。积极探索和推进社会公益类、行政执行类事业单位改革。同时，加快引进竞争激励机制，推进以聘用制为核心的事业单位人事制度改革，实行多元化的分配方式，逐步建立符合事业单位发展规律的管理模式和薪酬制度。

同步推进其它各项改革。组织实施市级和县区政府机构改革，调整完善机构设置，规范政府部门职能。认真贯彻国务院关于投资体制改革的决定，落实企业投资自主权，充分调动社会多元投资积极性。继续深化财政体制改革，调整和优化财政支出结构，增加公共财政支出。支持金融机构深化改革，做好城市商业银行增资扩股工作，着手组建农村合作银行，积极引进股份制银行来我市设立分支机构。巩固农村税费改革成果，进一步规范农业税免征工作，努力化解县乡财政困难。加快推进水利工程管理体制和产权制度改革。继续探索和实行户籍管理制度改革。

（三）加快现代加工制造业基地建设步伐，进一步提升工业发展水平。围绕新一轮产业升级和结构调整，积极实施质量兴市战略，以骨干企业和优势产品为重点，加大工业投入，壮大企业规模，实现工业经济总量的快速扩张。

全力推动马钢实现跨越式发展。支持马钢提前实施“十一五”发展建设规划，开工建设500万吨薄板带项目，确保2007年建成投产，早日建成具有较强国际竞争力的现代企业集团。继续抓好1000万吨钢配套技术改造，建成小H型钢、2号热镀锌生产线，确保冷轧薄板、彩涂板等项目达产达效，马钢产能力争跃上1000万吨钢新台阶，销售收入超过300亿元。支持马钢启动新一轮矿山发展规划，加快推进后备矿山建设，积极参与国内外矿山、煤炭资源的合作开发，加强与铁路、港口等物流系统的战略合作，着力构筑资源安全保障体系。

集中力量实施一批重点工业项目。加快山鹰30万吨造纸、雨润食品等项目实施进度，确保中橡2万吨新工艺炭黑、蒙牛冰淇淋等一批重点项目年内竣工投产。积极扶持华菱公司发展，引导企业抢抓发展机遇，迅速做大做强。加快国家863新材料产业化基地建设步伐，组织实施磁材、环保、化工等新材料产业项目，不断提高高新技术产业对经济发展的贡献份额。加大对专精特新中小企业的扶持力度，支持企业加快技术改造，培育一批对行业发展有带动作用的骨干企业，形成企业梯次发展格局。全年力争新增规模以上工业企业60户以上，规模以上工业增加值增长20%，销售收入净增100亿元以上。同时，加大对经济运行的调节力度，着力化解加快发展的“瓶颈”制约，推动全市工业经济快速健康发展。

加紧编制和启动“十一五”规划。紧紧围绕“三步走、翻三番”战略目标，编制完成国民经济与社会发展“十一五”规划纲要，以及工业、农业和农村经济、高新技术、综合交通等17个专项规划，切实发挥规划在国民经济和社会发展中的龙头作用。继续推进二电厂二期扩建、机场高速、城际铁路等重大项目前期工作，争取一批发展基础好、符合产业政策的大项目列入国家和省“十一五”规划，为后续发展提供项目支撑。

（四）继续推进基础设施建设，不断完善城市现代化支撑服务体系。在继续拓展城市外延的同时，突出抓好老城改造和建成区配套完善，积极发展第三产业，建设优美城市，努力适应经济社会发展和改善人民生活的需要。

加大城市建设和管理力度。新建一批城市道路，改造扩建205国道湖北路至采石河路段等道路，维修一批城市干道和二级道路，全年新建、改造城市道路25公里。实施城市入口和主要路段绿化亮化工程，进一步美化城市形象。建成第四水厂、开发区污水处理厂和王家山污水处理厂扩建主体工程，全面完成天然气利用工程，加紧建设500千伏开关站、220千伏江东变等电力设施。建成马芜高速公路，推进6号码头扩建工程建设，力争长江大桥早日开工，适时启动宁芜复线改造。切实加强城市管理。完善相对集中行政处罚权工作，继续整治违法建筑、占道经营、乱涂乱贴等违法违规行为，健全和落实城市管理的长效机制。强化道路交通管理，提高城市主干道通行能力，实现近期缓解、远期畅通的目标。加快建设管理有序、服务完善、环境优美、文明祥和的新型社区，充分发挥社区在城市管理中的基础性作用。

全面落实最严格的土地管理制度。按照规划要求，合理确定各类用地规模和区域布局，严格依法保护基本农田。抓紧实施土地复垦计划，加大土地整理和耕地开垦力度，确保全市耕地占补平衡。加强土地利用计划管理，着力盘活存量土地，严禁土地未批先用或征而不用。进一步完善征地补偿办法和城市房屋拆迁政策，健全征地程序，加快安置房建设步伐，维护被征地农民、城市拆迁户和用地者的合法权益。大力宣传、认真贯彻土地管理法律法规，切实做到珍惜土地资源，合理利用每一寸土地。

扎实抓好生态市建设。把创建国家环保模范城市作为生态市建设的阶段性目标，以生态省综合示范基地建设为契机，全面落实创建工作各项部署，力争一次性通过考核验收。严格执行建设项目与环境保护“三同时”制度，深化工业污染治理，坚决淘汰高污染、高能耗、工艺落后的设备和项目。加大环保执法监督力度，狠抓区域环境评价和环境污染突出问题的整治。积极发展循环经济，认真组织开展清洁生产试点工作，着力构建资源节约型经济体系。切实加强水资源保护，加快推进采石河综合整治工程，抓紧实施大公圩等水环境综合治理。深入开展矿区生态建设，大力发展生态农业，积极创建国家环境优美乡镇、生态村和生态示范户，不断提高城乡可持续发展能力。

加快发展现代服务业。围绕打造休闲旅游基地，全面实施旅游总体规划，加快采石风景区周边环境改造，启动濮塘风景区开发与建设。创??游业快速发展。进一步拓展商贸流通领域，大力发展连锁经营、物流配送、电子商务等新型业态。有选择地引进大型商贸企业，加快建设大华购物中心和新都商品专业批发市场，建成市中心步行街，改造解放路商业街，完善商业服务体系。继续规范房地产市场，促进房地产业健康发展。全年完成房地产开发投资24亿元，新开工面积180万平方米，竣工100万平方米以上。支持金融机构调整信贷结构，增加对骨干企业、重点项目的贷款投放，为中小企业和农村经济发展提供更多的资金支持。积极培育和规范会计、律师、咨询等中介服务组织。

（五）坚持统筹城乡发展，推动县区经济综合实力不断迈上新台阶。继续加大对“三农”的支持力度，保持农业和农村发展的良好势头，不断壮大县区经济规模，实现城乡协调发展。

进一步强化农业基础地位。按照“各项支农措施力度只能加大，不能减弱”的要求，全面落实中央和省、市各项支农惠农政策，切实保护好广大农民的生产积极性。围绕建设绿色食品基地，扎实推进农业结构调整，加快水产品、优质粮油、苗木花卉等六大优势农产品生产基地建设，形成区域化布局、规模化生产和标准化管理，提高农产品市场竞争能力。发挥骨干龙头企业的牵动作用，大力发展农业产业化经营，引导企业与农户建立稳固的利益联结机制，促进农产品就地转化增值。强化农产品现代营销体系建设，不断扩大农产品市场供应份额。加大农业招商力度，力争全年引进项目资金4亿元以上，推动“三资”农业向更高层次发展。继续增加农业科技投入，加强农业科技推广体系建设。

加快县区二三产业发展。加强对县区经济发展的指导，从规划、用地、产业转移等方面支持县区优化经济结构，调整产业布局。县区要以发展民营经济为抓手，充分发挥比较优势，按照用地集约、工业集中、产业集聚和环境保护的要求，引进和实施一批新项目，增强发展后劲。积极打造乡镇工业经济板块，重点培育博望刃模具和机床、黄池食品等产业集群，发挥产业规模效应。鼓励发展村级经济，加快小康村建设步伐。加大农村创业主体培育力度，支持发展农民专业合作经济组织、农村经纪人队伍，引导农村富余劳动力向城市和小城镇转移，拓展农民增收渠道。

不断改善县区加快发展的基础条件。推动市县相关干道对接，续建围乌公路、新黄公路，开工建设澄湖公路、霍薛公路和部省通达工程，全年新增农村公路120公里，进一步改善农村交通条件。支持三区加强基础设施建设，加快居民小区配套改造。扎实推进小城镇建设，提升重点镇综合服务功能，全年完成投资6亿元以上。继续加强农田水利基本建设，组织实施万亩以上圩口堤防和病险水库、涵闸除险加固工程，大规模兴修农村当家塘，增强城乡防洪保安和抗旱减灾能力。

三、大力推进社会全面协调发展

牢固坚持科学发展观，在加快经济发展的同时，重视发展科技教育和社会事业，加强精神文明建设，努力促进经济、社会和人的全面发展。

（一）继续实施科教兴市战略。深化科技体制改革，着力整合科技资源，促进科技成果转化，为经济社会发展提供有力支撑。加快高新技术创业服务中心后续工程建设，力争863新材料产业园主体工程年内完工。进一步拓展产学研合作渠道，鼓励企业研发具有自主知识产权的核心技术，增强自主创新和产业发展能力。继续加强信息基础设施建设，推进省级制造业信息化重点市实施步伐，提高信息技术在经济发展中的应用水平，加快建设“数字马鞍山”。切实把教育放在优先发展的位置。巩固“两基”教育成果，优化调整中小学布局，提高高中阶段教育比重，增强职业教育市场适应能力。组织开展对口支教活动，鼓励城市示范学校与农村中小学合作办学，探索建立寄宿学校，继续改造农村中小学危房，改善农村办学条件。加快安工大东校区二期工程建设，扩大师范高等专科学校办学规模，力争引进1—2所国内知名大学来我市办学，促进高等教育快速发展。实现安徽工贸技师学院预期办学目标，推进安工大职业技术学院、安徽冶金科技职业学院扩建工程，努力为经济社会发展培养更多的技术技能型人才。完善多渠道教育投入体制，引导和促进民办教育健康发展。鼓励市民参加各种形式的继续教育，推动学习型城市建设迈上新台阶。大力实施人才强市战略。全面落实人才工作的各项政策，努力形成育才、聚才、用才的新机制和新优势，充分发挥各类人才在推进经济社会发展中的重要作用。

（二）统筹发展各项社会事业。以深化文化体制改革为动力，大力发展公益性文化事业和经营性文化产业，繁荣文化市场，满足多层次的文化需求。开工建设大剧院、博物馆和新图书馆，加强基层文化设施建设。力争首届中国诗歌节申办成功。强化政府公共卫生管理职能，建成市传染病专科医院，重视做好艾滋病、结核病和血吸虫病等重大传染病防治工作。加强基层公共卫生体系建设，全面实施新型农村合作医疗制度，逐步提高农村医疗保障水平。广泛开展群众体育活动，争取在竞技体育上实现新的突破。坚持国防建设与经济建设协调发展，加强国防动员和民兵预备役建设，加速推进军事斗争准备。巩固和发展军政军民团结，力争早日进入全国双拥模范城市行列。积极推进广播电视数字化进程，巩固和发展农村广播电视事业。继续强化人口与计划生育工作，综合治理出生人口性别比偏高问题。完成第一次全国经济普查。做好第六届村委会换届工作。进一步推进民族、宗教、对台、侨务等工作，积极发展新闻出版、气象、抗震、科普、地方志、档案等各项事业，切实维护妇女、儿童、老年人的合法权益，促进残疾人事业和关心下一代工作的新发展。

（三）努力提升社会文明程度。以创建全国文明城市为龙头，广泛开展各种形式的精神文明创建活动。对照《全国文明城市测评体系》标准和要求，抓紧做好攻坚达标工作，力争进入首批全国文明城市行列。深入创建“八百里皖江文明长廊”，整体推进文明行业、文明单位、文明社区、文明村镇等群众性创建工作，促进城乡同创共建。广泛开展各具特色的诚信教育活动，围绕构建政府、企业、个人三大信用主体，落实好建设“信用马鞍山”的各项工作措施。继续整治校园及周边秩序，为师生创造良好的教学环境。深入开展未成年人思想道德实践活动，构建学校、家庭、社会三位一体的工作网络，提高未成年人的思想道德素质。

（四）切实提高人民生活水平。坚持以人为本、富民为先，使改革开放和经济发展的成果惠及城乡广大人民群众。继续做好就业再就业工作。扶持发展劳动密集型产业和中小企业，努力增加就业岗位。进一步落实促??职业、自主创业。加大就业培训力度，重点加强对失业人员、被征地农民和农村富余劳动力的职业技能培训，增强劳动者就业、创业和职业转换能力。全年力争新增就业岗位2.1万个，其中失业人员再就业1万人，“4050人员”再就业2000人，农村新增劳务输出2万人。深化安置方式改革，积极稳妥地做好军转干部和退役士兵安置工作。继续扩大养老、失业、医疗保险覆盖面，加快推进工伤、生育保险，启动实施被征地农民养老保障制度。加强和改进社会保险稽核，实现社会保险费应收尽收。不断完善城镇居民最低生活保障制度，扩大农村低保工作试点。实施城乡特困家庭大病救助、义务教育和就业援助制度，逐步健全社会救助体系。认真贯彻国务院《劳动保障监察条例》，健全企业职工维权机制，督促用人单位落实最低工资标准制度，严厉查处拖欠农民工工资行为。继续实施廉租房和经济适用房制度，完成老城区旧房改造15万平方米，进一步改善困难群众的居住条件。

（五）扎实做好维护稳定工作。以构建社会主义和谐社会为目标，大力创建“平安马鞍山”。高度重视解决新形势下的人民内部矛盾，切实改进信访工作方法，继续实行领导干部下访督查制度，建立健全社会协调机制。依法妥善处置群体性事件，积极做好社会特殊群体的稳定工作，最大限度地预防和化解社会矛盾。全面落实社会治安综合治理各项措施，扎实推进社会治安防控体系建设。严惩各类严重刑事犯罪活动，坚决打击黄、赌、毒等社会丑恶现象。加强法制宣传教育，深入开展“四五”普法活动，不断提高全民法律素质和全社会法治化水平。严格落实安全生产责任制和责任追究制，加快完善消防、交通安全设施，强化重大安全事故隐患整治，保障人民群众生命财产安全。高度重视社会预警体系建设，完善重大自然灾害、社会安全等突发事件应急处置预案，努力提高政府应对公共危机的能力。进一步加强收费监管，稳定市场物价。继续整顿和规范市场经济秩序，重点加强对直接关系人民群众身体健康和生命安全的食品、药品专项整治，严厉打击各种制假售假行为，切实维护公众消费安全。

四、不断提高政府行政能力

在加速实现“两个率先”的伟大实践中，各级政府系统要深入贯彻党的十六届四中全会精神，按照中央和省、市委的统一部署，积极组织开展保持党员先进性教育活动；按照加强执政能力建设的要求，着力提升干部队伍素质，不断提高政府施政水平。

（一）大力推进依法行政。认真贯彻国务院《全面推进依法行政实施纲要》，逐步完善各项具体办法和配套措施，以创新精神推动依法行政。按照《行政许可法》的要求，健全行政服务中心功能，积极推进行政许可权的相对集中行使。认真执行人大及其常委会的决议和决定，高质量办理人大代表议案和建议批评意见，自觉接受人大及其常委会的法律监督和工作监督。支持政协履行政治协商、民主监督和参政议政职能，加强与各民主党派、工商联、人民团体的联系，主动接受社会各方面的监督。继续推进政务公开，完善新闻发布制度，提高政府工作透明度。进一步落实行政执法责任制和执法过错责任追究制，扎实做好行政复议、行政应诉、行政赔偿工作，做到有权必有责、用权受监督、违法要追究、侵权要赔偿。

（二）着力提高行政效能。深入推进行政审批制度改革，规范行政审批行为，更好地服务经济发展、服务人民群众。积极创新行政审批方式，完善工商注册、并联审批告知承诺制，进一步提高审批效率。加快电子政务建设，推进政府管理创新，降低行政成本。出台实施机关效能建设工作暂行规定，推行行政问责制，认真查处行政效率低下和行政不作为、乱作为问题，不断增强机关工作人员的责任意识和服务意识。广泛开展机关作风和效能评议，把评判政府服务质量和工作绩效的权力交给群众、交给企业、交给投资者，促进政风的进一步好转。继续完善目标管理责任制，加大考核奖惩力度，提高工作落实水平，推动政府工作全面提速增效。

（三）不断完善决策机制。决策是政府工作的首要环节，提高决策水平是做好政府工作的前提条件。要按照决策科学化、民主化和行政为民的要求，进一步明确决策原则和范围，完善决策程序，切实防止和克服决策的随意性和盲目性。在决策过程中，加强调查研究，坚持发扬民主，广泛集中民智。对涉及经济社会发展全局的重大事项，决策前广泛征求意见，充分进行协商和协调；对专业性、技术性较强的重大事项，认真进行专家论证、技术咨询和决策评估，注重听取市政府顾问和咨询委专家的意见；对关系群众切身利益的重大事项，严格实行公示、听证制度，使决策充分体现群众意愿。建立健全决策信息反馈和评估机制，为不断完善决策机制提供科学依据。

（四）继续加强廉政建设。认真落实政府廉政工作任务和领导干部廉政建设责任制，深入开展述职述廉活动，进一步规范从政行为。继续完善建设工程招投标、经营性土地使用权出让、产权交易、政府采购、财政预决算等制度，严格实行收支两条线和国库集中支付，大力推进政府性投资“代理制”，从源头上预防腐败现象发生。加大城市道路、公路和水利工程的执法监察力度，强化对重大项目建设、重要财政支出的监督和审计，继续抓好领导干部离任审计和任期审计，试行审计结果公告制，严肃查处各种违法违纪案件。高度重视解决群众反映强烈的突出问题，坚决纠正损害群众利益的不正之风。政府全体工作人员要牢固树立廉洁从政、勤政为民的思想，保持艰苦奋斗、密切联系群众的作风，努力建设廉洁、勤政、务实、高效的政府。

各位代表，马鞍山正处在跨越发展的历史新时期。让我们紧密团结在以胡锦涛同志为总书记的党中央周围，在省委、省政府和市委的坚强领导下，高举邓小平理论和“三个代表”重要思想伟大旗帜，以饱满的热情、昂扬的斗志、创新的精神、扎实的工作，为加速实现“两个率先”目标作出新的更大贡献！
